# Activating FLT3 Mutants Show Distinct Gain-of-Function Phenotypes In Vitro and a Characteristic Signaling Pathway Profile Associated with Prognosis in Acute Myeloid Leukemia

**DOI:** 10.1371/journal.pone.0089560

**Published:** 2014-03-07

**Authors:** Hanna Janke, Friederike Pastore, Daniela Schumacher, Tobias Herold, Karl-Peter Hopfner, Stephanie Schneider, Wolfgang E. Berdel, Thomas Büchner, Bernhard J. Woermann, Marion Subklewe, Stefan K. Bohlander, Wolfgang Hiddemann, Karsten Spiekermann, Harald Polzer

**Affiliations:** 1 Department of Internal Medicine III, University Hospital Munich, Ludwig-Maximilians-University Munich, Germany; 2 Clinical Cooperative Group Leukemia, Helmholtz Center Munich, Germany; 3 German Cancer Consortium (DKTK), Heidelberg, Germany; 4 German Cancer Research Center (DKFZ), Heidelberg, Germany; 5 Department of Biochemistry, Gene Center, Ludwig-Maximilians-University Munich, Germany; 6 Department of Medicine A, Hematology, Oncology and Pneumology, University Muenster, Germany; 7 German Society of Hematology and Oncology, Berlin, Germany; 8 Clinical Cooperative Group Immunotherapy, Helmholtz Center Munich, Germany; 9 Department of Molecular Medicine and Pathology, University of Auckland, New Zealand; Josep Carreras Leukaemia Research Institute, University of Barcelona, Spain

## Abstract

About 30% of patients with acute myeloid leukemia (AML) harbour mutations of the receptor tyrosine kinase FLT3, mostly internal tandem duplications (ITD) and point mutations of the second tyrosine kinase domain (TKD). It was the aim of this study to comprehensively analyze clinical and functional properties of various FLT3 mutants.

In 672 normal karyotype AML patients *FLT3*-ITD, but not *FLT3*-TKD mutations were associated with a worse relapse free and overall survival in multivariate analysis. In paired diagnosis-relapse samples *FLT3*-ITD showed higher stability (70%) compared to *FLT3*-TKD (30%). *In vitro*, FLT3-ITD induced a strong activating phenotype in Ba/F3 cells. In contrast, FLT3-TKD mutations and other point mutations – including two novel mutations – showed a weaker but clear gain-of-function phenotype with gradual increase in proliferation and protection from apoptosis. The pro-proliferative capacity of the investigated FLT3 mutants was associated with cell surface expression and tyrosine 591 phosphorylation of the FLT3 receptor. Western blot experiments revealed STAT5 activation only in FLT3-ITD positive cell lines, in contrast to FLT3-non-ITD mutants, which displayed an enhanced signal of AKT and MAPK activation. Gene expression analysis revealed distinct difference between *FLT3*-ITD and *FLT3*-TKD for STAT5 target gene expression as well as deregulation of *SOCS2*, *ENPP2*, *PRUNE2* and *ART3*.

*FLT3*-ITD and *FLT3* point mutations show a gain-of-function phenotype with distinct signalling properties *in vitro*. Although poor prognosis in AML is only associated with FLT3-ITD, all activating *FLT3* mutations can contribute to leukemogenesis and are thus potential targets for therapeutic interventions.

## Introduction

The Fms-like tyrosine kinase 3 (FLT3) plays an essential role in hematopoiesis, driving differentiation of early myeloid and lymphoid lineages, but being down-regulated at later stages. Its expression is usually tightly restricted to early progenitor cells and deregulation of the FLT3 receptor plays a major role in the pathogenesis of leukemia [1], [2]. Mutations of *FLT3* are found in 30% of acute myeloid leukemia (AML) cases, making it the most frequently mutated tyrosine kinase in this otherwise heterogeneous group [2], [3]. The most common alterations, internal tandem duplications (ITD) in the juxtamembrane (JM) domain of the FLT3 receptor are associated with poor prognosis, with respect to event free survival, relapse rate and overall survival [4], [5]. Disruption of the FLT3-JM through ITD results in a loss of its autoinhibitory function and conveys ligand-independent phosphorylation and activation of FLT3 [1], [6]. A second class of recurring mutations are gain-of-function mutations at the amino acids (AA) 835/836 in the second tyrosine kinase domain (TKD) [3], [7], [8]. In addition to these, rare activating point and length mutations have been described [9]–[13]. Although *FLT3-*ITD strongly influence disease-phenotype and prognosis, the genotype-phenotype relationship is not so clear for *FLT3* point mutations, which are not considered to be an independent prognostic factor. However, acquired FLT3 point mutations can induce resistance to FLT3 tyrosine kinase inhibitors in AML patients [14]–[16]. The results in terms of classification into gain-of-function mutations and mutations without activating phenotype in the *FLT3* gene *in vitro* and *in vivo* are inconsistent [4], [5], [7], [9], [17].

Here we analyzed clinical data regarding different parameters and outcome variables with respect to *FLT3* mutation status and investigated the influence of various *FLT3* mutations in a comparative setting. We present data showing that *FLT3* point mutations are gain-of-function mutations which induce a range of changes in cell growth and apoptosis susceptibility *in vitro*. These mutations differ considerably from *FLT3*-ITD with respect to prognosis, mutation stability at relapse and signaling patterns *in vitro* and *in vivo*.

## Materials and Methods

### Analysis of Clinical Outcome

The independent prognostic effect of *FLT3*-WT*, *FLT3*-ITD, *FLT3*-TKD or both *FLT3* mutation types on overall survival (OS), relapse free survival (RFS) and complete remission (CR) was evaluated in 672 of 802 CN-AML patients. The asterisk denotes the fact that only the mutational hotspots, and not the entire FLT3 gene, were sequenced. These patients were enrolled in the AMLCG99 trial [18]. The AMLCG clinical trial was approved by the local institutional review boards of all participating centers and informed consent was obtained from all patients in accordance with the Declaration of Helsinki. 329 patients were female (329/672, 58.3%). The median age was 60 years (range: 16–85 years). 554 patients (82.4%) had *de novo* AML, 70% had a performance status according to Eastern Cooperative Group (ECOG) of ≤1. 19% underwent allogeneic transplantation. Mutations of *NPM1*, biallelic *CEBPA* mutations (bi*CEBPA*) and partial tandem duplications of the *MLL* gene (*MLL*-PTD) were present in 53% (329/620), 5% (29/638) and 7% (48/645) of cases, respectively.

The outcome parameter OS was calculated from the time of randomization to death from any cause or to the latest follow-up date. RFS was determined for responders from the first day of CR until relapse or death without relapse. In patients who had undergone allogeneic transplantation, survival times were censored at the timeof transplantation. Kaplan Meier estimates of survival as well as univariable and multivariable Cox regression analyses were performed for OS and RFS. Factors included in the model were: age, performance status (ECOG), de novo AML, white blood count (WBC), platelet count, hemoglobin level, lactate dehydrogenase (LDH) level, bone marrow (BM) blasts, mutation status of *NPM1*, *FLT3*, *MLL-PTD* and *CEBPA*. Due to correlation between *NPM1* and *FLT3*-ITD, an interaction term was included, which was 1 when both mutations were present and 0 if either *NPM1* or *FLT3*-ITD were present or both were WT*. Independent prognostic factors were identified with an exclusion significance level of 5%. Statistical analyses were performed using SPSS version 20.0 (SPSS Inc., Chicago, IL).

### Analysis of FLT3 Stability

The stability of *FLT3* mutationsduring the course of the disease was evaluated in 156 patients (out of 352 patients with relapsed AML) with available bone marrow aspirates at diagnosis and at relapse that were screened for *FLT3*-ITD and *FLT3*-TKD mutations as previously described [19], [20]. *FLT3*-ITD mRNA levels at diagnosis and relapse were calculated a following: (*FLT3*-ITD/*FLT3*-WT)/(*FLT3*-ITD/*FLT3*-WT+1) [21]. In case of absence of hotspot mutations the receptor mutation status was named WT*. 74 of 156 patients were female. The median age was 60 years with a range of 21 to 90 years. 130 (85.5%) patients had *de novo* AML, 16 (10.5%) sAML and 5 (3%) t-AML. In five patients the origin of AML was not reported. The majority of patients had an intermediate karyotype (n = 102; cytogenetically normal (CN-AML) n = 92), eight patients showed a favorable karyotype, and 46 had an adverse type at first diagnosis [22]. The majority of patients (76.3%) had been treated within AMLCG studies (AMLCG92 n = 3; AMLCG99 n = 98; AMLCG2004 n = 9; AMLCG2008 n = 6; others: n = 3) [18], [23], [24]. The AMLCG clinical trials were approved by the local institutional review boards of all participating centers and informed consent was obtained from all patients in accordance with the Declaration of Helsinki.

### Plasmids and Antibodies

DNA constructs and vectors were used as described before [9], [25]. FLT3-I867S and FLT3-D839G constructs were generated using QuikChange II XL Site-Directed Mutagenesis Kit (Agilent, Santa Clara, CA). Denotation: W78: ITD1, Npos: ITD2, W51: ITD3. Figure S1 displays the locations and insertions respectively substitutions of the analyzed mutations.

The following antibodies were used: FLT3 (S18) and goat anti-mouse secondary antibody from Santa Cruz Biotechnology (Santa Cruz, CA). AKT, MAPK, pSTAT5 (Tyr694), pAKT (Ser473) and pMAPK (Thr202/Tyr204) all from Cell Signaling Technology (Danvers, MA). STAT5 from R&D Systems (Minneapolis, MN). β-actin and goat anti-rabbit secondary antibody from Sigma-Aldrich (St. Louis, MO). CD-135-PE from Beckman Coulter (Brea, CA). IgG1 PE Isotype control from BD Pharmingen (BD Bioscience, Franklin Lakes, NJ).

### Cell Lines and Reagents

Murine Ba/F3 and WEHI-3B cell lines were obtained from Deutsche Sammlung von Mikroorganismen und Zellkulturen (DSMZ) (Braunschweig, GER). Phoenix Eco cells were purchased from Orbigen (San Diego, CA). Method of characterization can be obtained from the respective cell bank. Cells were cultured according to vendors' instructions.

Recombinant human FLT3 ligand (FL) was purchased from Promokine (Heidelberg, GER) and recombinant murine IL-3 from Immunotools (Friesoythe, GER). The inhibitor MK 2206 was obtained from Selleck Chemicals (Houston, TX).

### Transient Transfection of Phoenix Eco and Stable Transduction of Ba/F3 Cells

These experiments were carried out as described previously [26]. BaF3 cells were stable transduced with FLT3 plasmids including an EGFP-IRES site (MSCV-IRES-EGFP). Fluorescence activated cell sorting was performed to generate pure cell lines. cDNA of all generated cell lines were screened for the presence of ITD using a fragment length analysis after FLT3 amplification by PCR. Additionally presence of FLT3 mutations in the generated cell lines was confirmed by Sanger sequencing.

### Proliferation and Apoptosis Assays

Proliferation and apoptosis assays were performed as described before [9]. Cells were counted using Trypan blue exclusion, automated by Vi-CELL AS from Beckman Coulter (Brea, CA). Analysis of flow cytometry data was performed using Windows Multiple Document Interface for Flow Cytometry 2.8. (WinMDI; Joe Trotter).

### Western Blot Analysis

Experiments were performed as described before [26]. Proteins were visualized by chemiluminescence using ECL Plus Western Blot Detection Kit (GE Healthcare, Chalfont St.Giles, UK). Semi-quantitative analysis of protein bands was performed using Image J 1.44 (Wayne Rasband, National Institutes of Health). Values indicate ratio of phosphoylated to total protein expression levels.

### ELISA

PathScan Phospho-FLT3 (Tyr591) Chemiluminescent Sandwich ELISA, Cell Signaling Technology (Danvers, MA) was used according to the manufacturer's instruction.

### Statistical Analysis of *In Vitro* Data

Statistics were implemented with SigmaPlot 11.0 and two sided t-test as well as Mann-Whitney Rank Sum test were used. Heatmap was created using R Development Core Team (2011). R: A language and environment for statistical computing. R Foundation for Statistical Computing, Vienna, Austria.ISBN 3-900051-07-0.Retrieved fromhttp://www.R-project.org/.

### Gene Expression Analysis

A subgroup of patients included in the gene expression data set GSE37642 was analyzed [27]. All patients were enrolled in the AMLCG-99 trial [18]. The subgroup was selected according to the following characteristics: CN-AML and information on *FLT3*, *MLL*, *NPM1* and *CEBPA* mutation status. In case of absence of hotspot mutations the receptor was named WT*. Table S1 shows detailed patient characteristics.

Pretreatment bone marrow samples were analyzed using Affymetrix HG-U133 A/B and 2.0 plus microarrays (Affymetrix, Santa Clara, CA) following standard protocols [27]. For probes to probe set annotation we used custom chip definition files (CDFs) based on GeneAnnot version 2.0, synchronized with GeneCards Version 3.04 (available at http://www.xlab.unimo.it/GA_CDF) [28]. These CDFs decrease the total number of probe sets (one probe set per gene), and potentially increases the specificity of the analyses by eliminating cross-hybridizing probes (probes are restricted by sequence specificity). Data normalization was performed as described previously [29]. 17389 probe sets present both on the A, B chips and the 2.0 plus chips were included in the analysis.

Gene set enrichment analysis (GSEA) was performed with GSEA software (MIT) to assess significant changes in gene expression levels [30]. The GSEA was run with 1,000 permutations and compared with Signal2Noise to the “c3_tft” collection from the Molecular Signatures Database MsigDB 3.0 (http://www.broadinstitute.org/gsea/msigdb/index.jsp) consisting of 615 gene sets that share a transcription factor binding site defined in the TRANSFAC (version 7.4, http://www.gene-regulation.com) database and the “c2_kegg” collection consisting of 186 gene sets from the Kyoto Encyclopedia of Genes and Genomes data base (KEGG). The minimum size of tested gene sets was 15 and the maximum was 500. The Linear Models for Microarray Data (Limma) package was used to compute differentially expressed probe sets based on the FLT3-status. All statistical analyses were performed using the R 2.11.0 software and routines from the biostatistics software repository Bioconductor.

## Results

### FLT3-TKD Mutations Are Associated with Superior RFS and OS Compared to FLT3-ITD

The impact of the *FLT3* mutation status was evaluated in 672 patients with CN-AML. The majority of patients were *FLT3*-WT* (64.7%). *FLT3*-ITD and *FLT3*-TKD mutations were present in 28.6% and 5.5%, respectively. 1.2% of patients had both types of mutations. 128 of 673 patients (19%) underwent allogeneic transplantation. The proportion of allogeneic transplanted patients in the *FLT3*-WT* (n = 76/436; 17.4%), *FLT3*-ITD (n = 42/192; 21.9%) and *FLT3*-TKD (n = 8/37; 21.6%) group were not significantly different (p = 0.556).

**Figure 1:**
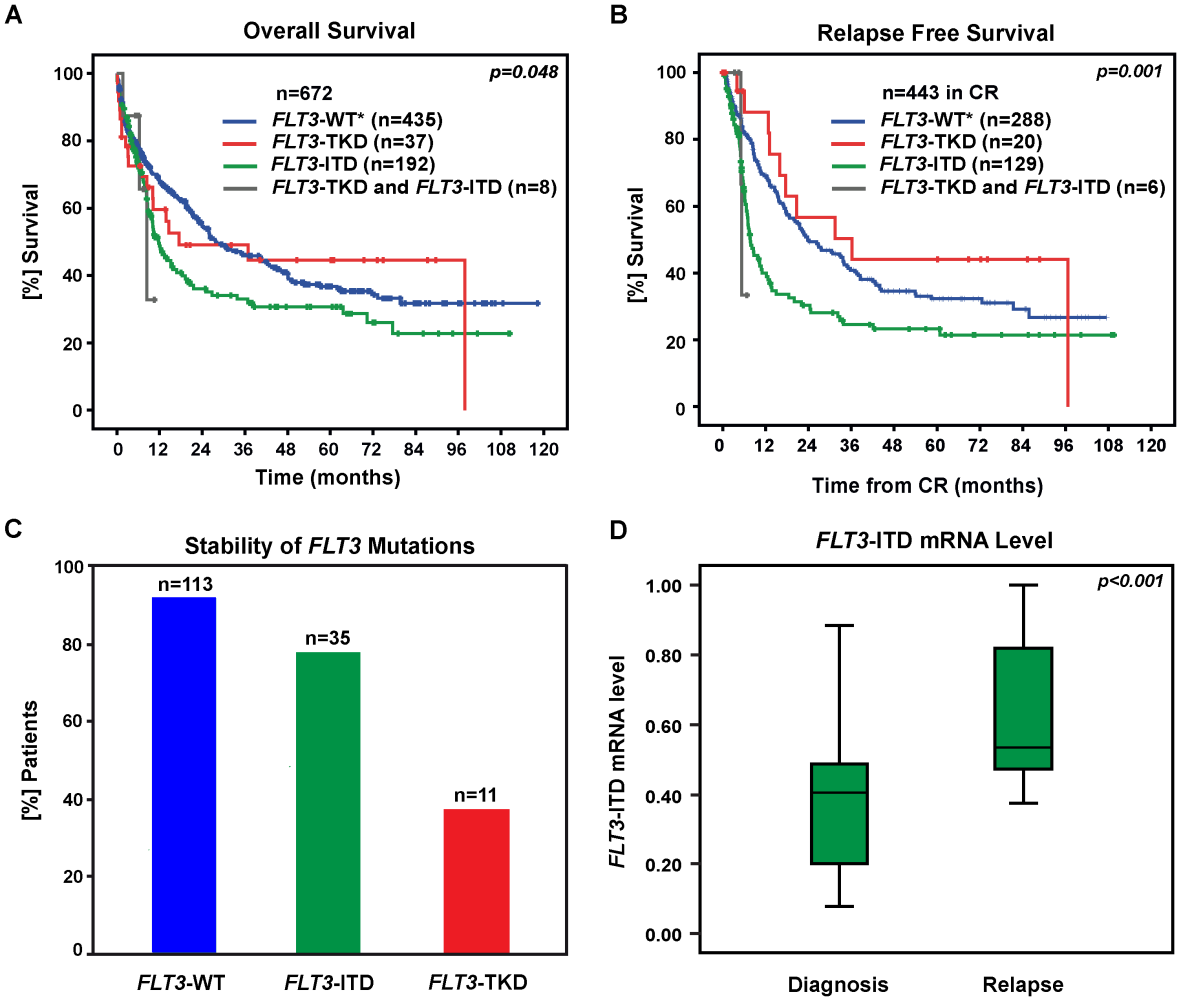
Prognostic impact and genetic stability of FLT3-ITD and FLT3-TKD mutations in AML. (A) OS in 672 patients was significantly different between *FLT3*-mutated patients compared to patients with *FLT3*-WT*. OS in patients with *FLT3*-TKD mutations was not different compared to *FLT3*-WT* expressing patients (HR: 0.8, 95% CI: 0.7–1.7). Patients with a *FLT3*-ITD showed a significantly worse OS compared to those with *FLT3*-WT* (HR: 1.4, 95% CI: 1.1–1.8). There was no significant difference in the OS of patients with both *FLT3*-ITD and *FLT3*-TKD mutation compared to those with *FLT3*-ITD (HR 1.1, 95% CI: 0.3–3.4). (B) RFS in 443 patients in first complete remission was significantly different between *FLT3*-mutated patients compared to patients with *FLT3*-WT*. Patients with *FLT3*-TKD mutation had no significant superior RFS compared to *FLT3*-WT* (HR: 0.8, 95% CI: 0.4–1.4). Patients with a *FLT3*-ITD showed a significantly worse RFS compared to those with *FLT3*-WT* (HR: 1.7, 95% CI: 1.3–2.2). There was no significant difference in the RFS of patients with both *FLT3*-ITD and *FLT3*-TKD mutation compared to those with *FLT3*-ITD (HR 1.2, 95% CI: 0.3–5.0). (C) 10 of 113 patients positive for *FLT3*-WT* at diagnosis acquired a *FLT3* mutation at relapse. 27 of 35 (77%) patients with a *FLT3*-ITD at initial diagnosis displayed a *FLT3*-ITD at relapse, whereas the majority of patients with a *FLT3*-TKD mutation (7/11; 63%) lost this mutation at relapse. (D) *FLT3*-ITD mRNA levels in 20 patients with a *FLT3*-ITD at diagnosis and at relapse were calculated. *FLT3*-ITD mRNA levels were calculated as following: (*FLT3*-ITD/*FLT3*-WT)/(*FLT3*-ITD/*FLT3*-WT+1). Median *FLT3*-ITD mRNA level was significantly higher at the time of relapse compared to first diagnosis (0.54 [range 0.37–1.00] versus 0.40 [range 0.08–0.88]; p<0.001, Wilcoxon test).

The median OS of patients with *FLT3*-WT* (n = 435), *FLT3*-ITD (n = 192), *FLT3*-TKD (n = 37) and both types of *FLT3* mutations (n = 8) was 27.7, 11.8, 17.5 and 8.4 months, respectively. Patients with *FLT3*-ITD displayed a significantly worse OS compared to patients expressing *FLT3*-WT* (Hazard Ratio (HR): 1.4, 95% confidence interval (CI): 1.1–1.8). In contrast, OS in *FLT3*-TKD mutation positive patients was not different compared to *FLT3*-WT* (HR: 0.8, 95% CI: 0.7–1.7). The last surviving patient under observation in the group of *FLT3*-TKD positive patients died of esophageal cancer. Patients who had both types of *FLT3* mutations showed a trend towards worse OS compared to patients with single *FLT3*-ITD mutation status (HR: 1.1, 95% CI: 0.4–3.3) (Figure 1A).

443 of 672 patients achieved a CR. The median RFS was 23.7 months for *FLT3*-WT* cases (n = 288), 7.7 months for *FLT3*-ITD (n = 129), 36.1 months for *FLT3*-TKD (n = 20) and 5.2 months for *FLT3*-ITD and *FLT3*-TKD mutation positive cases (n = 6). The presence of a *FLT3*-ITD was associated with a significantly shorter RFS compared to *FLT3*-WT* (HR: 1.7; 95% CI: 1.3–2.2). The positive effect of a *FLT3*-TKD mutation compared to FLT3-WT* status remained non-significant (HR: 0.8; 95% CI: 0.4–1.4). Patients with both types of *FLT3* mutations showed the worst RFS, which was not significantly different from RFS in patients with a single *FLT3*-ITD mutation (HR: 1.2; 95%: 0.3–5.0) (Figure 1B). To evaluate the independent prognostic effect of *FLT3*-ITD or *FLT3*-TKD on OS (n = 535) and RFS (n = 348) a multivariable Cox regression model was applied.

*FLT3*-ITD significantly worsened OS in *NPM1*-mutated patients (HR: 2.0; 95% CI: 1.4–3.0), whereas a *FLT3*-TKD had no significant effect on OS. *NPM1* mutations in the absence of *FLT3*-ITD (HR: 0.3; 95% CI: 0.2–0.4), and bi*CEBPA* mutations (HR: 0.3; 95% CI: 0.2–0.7) represented favorable prognostic factors of OS, whereas older age (HR: 1.05; 95% CI: 1.03–1.05) and high initial WBC (HR: 1.8; 95% CI: 1.4–2.2) were associated with unfavorable OS (Table 1).

**Table 1:** Multivariable analysis of prognostic factors for OS, RFS and CR.

OS n = 535						
					95% CI	
Parameter		Stratum	P	HR	Lower CL	Upper CL
*FLT3*-ITD		*NPM1*-WT*	0.280	0.76	0.46	1.25
*FLT3*-ITD		*NPM1*+	<0.001	2.05	1.39	3.03
*FLT3*-TKD	vs. *FLT3*-WT*		0.154	1.44	0.87	2.40
interaction *NPM1+/FLT3*-ITD			0.001	2.71	1.48	4.96
*NPM1*	pos. vs. neg.	*FLT3*-WT*	<0.001	0.30	0.20	0.44
bi*CEBPA*	vs. *CEBPA*-WT*/mo*CEBPA*		0.004	0.33	0.16	0.69
*MLL*-PTD	pos. vs. neg.		0.829	0.95	0.61	1.49
Age, yrs	+1 yr		<0.001	1.05	1.03	1.06
Performance Status, ECOG	2–4 vs 0,1		0.017	1.39	1.06	1.82
De novo AML	vs. non-de novo		0.788	0.96	0.68	1.33
WBC, ×10^6^/l	10-fold		<0.001	1.77	1.40	2.23
Platelets, ×10^6^/l	10-fold		0.525	0.89	0.62	1.27
Hemoglobin level, mg/dl	+1 g/dl		0.258	1.00	0.99	1.00
LDH, U/l	10-fold		0.874	1.00	1.00	1.00
BM blasts, %	+10%		0.827	1.00	0.99	1.01

RFS n = 348 in CR						
					95% CI	
Parameter		Stratum	P	HR	Lower CL	Upper CL
*FLT3*-ITD		*NPM1*-WT*	0.083	0.61	0.34	1.07
*FLT3*-ITD		*NPM1*+	<0.001	2.35	1.49	3.73
*FLT3*-TKD	vs. *FLT3*-WT*		0.923	1.04	0.51	2.10
Interaction *NPM1*+/*FLT3*-ITD			<0.001	3.89	1.94	7.78
*NPM1*	pos.vs. neg.	*FLT3*-WT*	<0.001	0.17	0.11	0.27
bi*CEBPA*	vs. *CEBPA*-WT*/mo*CEBPA*		<0.001	0.24	0.12	0.52
*MLL*-PTD	pos.vs. neg.		0.981	0.99	0.56	1.78
Age, yrs	+1 yr		0.024	1.01	1.00	1.03
Performance Status, ECOG	2–4 vs 0,1		0.099	1.32	0.95	1.84
De novo AML	vs. non-de novo		0.908	1.03	0.67	1.57
WBC, ×10^6^/l	10-fold		0.006	1.52	1.13	2.04
Platelets, ×10^6^/l	10-fold		0.284	0.80	0.53	1.20
Hemoglobin level, mg/dl	+1 g/dl		0.886	1.00	0.99	1.01
LDH, U/l	10-fold		0.533	1.00	1.00	1.00
BM blasts, %	+10%		0.200	1.01	1.00	1.01

CR n = 536						
					95% CI	
Parameter		Stratum	P	OR	Lower CL	Upper CL
*FLT3*-ITD			0.280	1.50	0.72	3.11
*FLT3*-TKD	vs. *FLT3*-WT*		0.154	0.65	0.30	1.45
Interaction *NPM1*+*/FLT3*-ITD			0.001	0.52	0.21	1.26
*NPM1*	pos.vs. neg.	*FLT3*-WT*	<0.001	3.08	1.77	5.36
bi*CEBPA*	vs. *CEBPA*-WT*/mo*CEBPA*		0.004	3.52	1.19	10.45
*MLL*-PTD	pos.vs. neg.		0.829	0.64	0.31	1.30
Age, yrs	+1 yr		<0.001	0.99	0.97	1.00
Performance Status, ECOG	2–4 vs 0,1		0.017	0.76	0.49	1.16
De novo AML	vs. non-de novo		0.788	1.59	0.94	2.70
WBC, ×10^6^/l	10-fold		<0.001	0.58	0.39	0.86
Platelets, ×10^6^/l	10-fold		0.525	1.80	1.25	2.59
Hemoglobin level, mg/dl	+1 g/dl		0.258	1.00	0.99	1.01
LDH, U/l	10-fold		0.874	1.06	0.53	2.13
BM blasts, %	+10%		0.827	1.00	0.99	1.01

To evaluate the independent prognostic impact of FLT3-ITD and FLT3-TKD on OS, RFS and CR, all candidate prognostic factors were included in the Cox regression model without selection of variables. The analyses were performed using 535 complete datasets of patients with regard to OS for the candidate prognostic factors. HR: Hazard Ratio; CI: confidence interval; CL: confidence limit.

Whereas the effect of a *FLT3*-TKD mutation was not significant with respect to RFS, the presence of *FLT3*-ITD in NPM1-mutated patients, significantly worsened RFS (HR: 2.4, 95% CI: 1.49–3.7). Older age (HR: 1.01; 95% CI: 1.00–1.03) and a high initial WBC (HR: 1.5; 95% CI: 1.1–2.04) had an independent negative impact on RFS. Independent prognostic factors leading to a significantly prolonged RFS included mutations of *NPM1* (HR: 0.2; 95% CI: 0.1–0.3) and bi*CEBPA* (HR: 0.24; 95% CI: 0.1–0.5). Performance status, *de novo* AML, platelet counts, hemoglobin level, LDH level, initial BM blasts and *MLL*-PTD did not significantly influence RFS (Table 1).

Analysis of *FLT3*-ITD mRNA level in a multivariate Cox regression model for OS and RFS revealed the same independent prognostic factors as analysis of *FLT3*-ITD in multivariate Cox regression models. The effect of a high *FLT3*-ITD mRNA level was restricted to *NPM1*-mutated patients (as shown previously; [21]). A high *FLT3*-ITD mRNA level had a stronger negative effect on OS and RFS (HR for OS: 4.7; 95% CI: 3.4–9.6 and HR for RFS: 5.4; 95% CI: 2.4–12.2) compared to the mere presence of a *FLT3*-ITD.

The logistic regression model for CR revealed *NPM1* (Odds Ratio (OR): 0.3; 95% CI: 0.2–0.6), and bi*CEBPA* mutations (OR: 0.3; 95% CI: 0.1–0.8), as well as initial WBC (OR: 1.7; 95% CI: 1.2–2.6) and platelet count (OR: 0.6; 95% CI: 0.4–0.8) as significant variables. Neither *FLT3*-ITD, nor *FLT3*-TKD mutations displayed a significant impact on CR (Table 1).

Similar results for OS and RFS with respect to *FLT3*-ITD and -TKD mutation status were obtained without censoring for allogenic transplantation and irrespective of age (patients <60 versus >60 years).

### Stability of FLT3 Mutations at Relapse

Out of 156 patients with available *FLT3* mutation status at diagnosis and relapse (matched pair samples), 113 were *FLT3*-WT*, eight patients expressed a single *FLT3*-TKD mutation and 31 displayed a single *FLT3*-ITD mutation. Three patients were positive for both types of mutation (ITD/TKD) and one patient had an ITD and a point mutation in the JM region (V592) of *FLT3*.

10 of 113 (9%) patients with *FLT3*-WT* at diagnosis acquired a *FLT3* mutation during relapse. 7 of 113 (6%) acquired a *FLT3*-ITD, 3 of 113 (3%) acquired a *FLT3*-TKD. 27 of 35 (77%) patients with a *FLT3*-ITD at initial diagnosis displayed a *FLT3*-ITD at relapse. None of those eight patients that had lost the *FLT3*-ITD expressed a *FLT3*-TKD mutation at relapse. In contrast, *FLT3*-TKD mutations present at diagnosis were lost in the majority of patients (n = 7/11; 63%) at the time of relapse (Figure 1C). 4 of 11 patients displayed the same *FLT3*-TKD mutation (D835) at diagnosis and relapse. One patient with a stable *FLT3*-TKD had acquired an additional *FLT3*-ITD at the time of relapse. In all three patients with simultaneous *FLT3*-ITD and *FLT3*-TKD mutation as well as *FLT3*-ITD and *FLT3*-V592 mutation at first diagnosis only the *FLT3*-ITD was present at relapse.

Patients with *FLT3*-ITD at diagnosis and relapse showed significantly higher *FLT3*-ITD mRNA levels at the time of relapse (Figure 1D).

### FLT3 Mutants Show a Broad Spectrum of Activating Phenotypes in Ba/F3 Cells

To clarify the potency to induce aberrant activation and signaling we analyzed eight different *FLT3* mutations: Three different FLT3-ITD constructs, FLT3-JM mutation V592A, common FLT3-TKD mutations D835Y and D835V as well as D839G and I867S in the second TKD (Figure S1). FLT3-D839G and -I867S were recently found in AML patients by our group during routine diagnostics but have not been functionally characterized before. The corresponding remission samples did not express these mutations showing that they are not rare germline variants.

**Figure 2:**
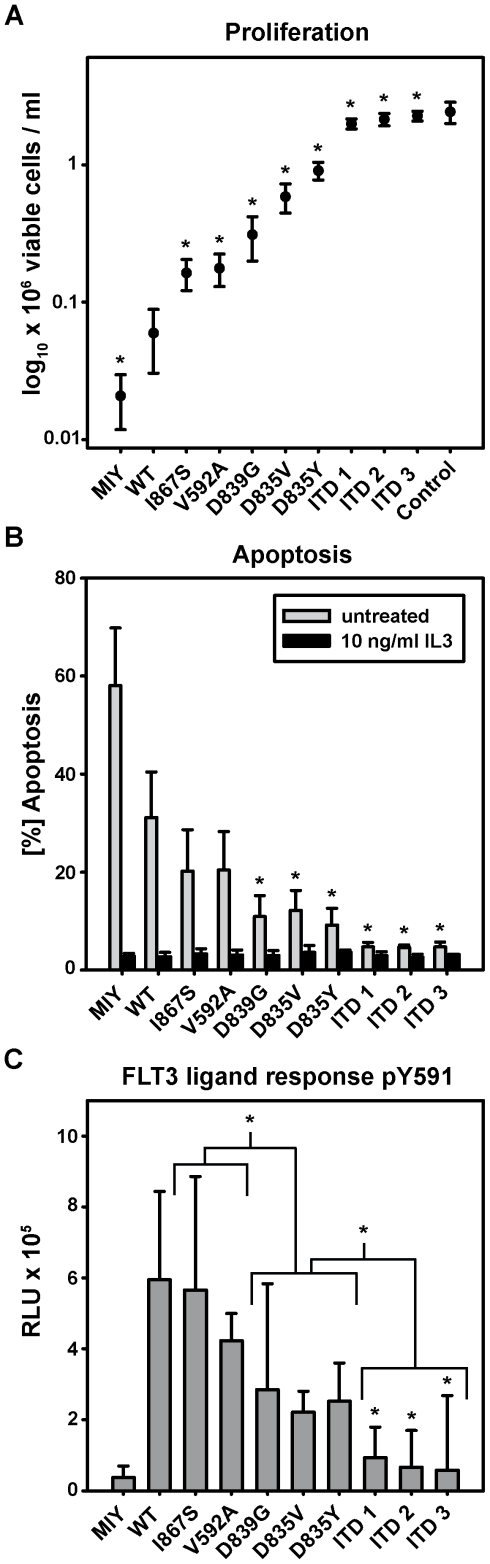
FLT3 mutants display a wide range of activating phenotypes in Ba/F3 cells. Ba/F3 cells stably transduced with indicated constructs were seeded at a density of 4×10^4^ cells/ml and cultured in presence and absence of 10 ng/ml IL-3. (A) Viable cells were counted after 72 h using trypan blue exclusion. Control indicates viable Ba/F3 MIY cells treated with 10 ng/ml IL-3. Values are expressed as mean +/− S.D. of nine independent experiments. (*) indicates significant higher growth compared to FLT3-WT expressing cells. (B) After incubation for 48 hours cells were stained with Annexin-V and 7-AAD. The percentage of apoptotic cells was determined using flow cytometry. Values are expressed as mean +/− S.D. of at least three independent experiments. (*) indicates significant higher apoptosis compared to FLT3-WT expressing cells. (C) Ba/F3 cells expressing FLT3 mutations were cultured without IL-3 for 24 hours. Cells were treated with 100 ng FLT3-ligand for 10 minutes prior to lysis to analyze phosphorylation of FLT3 receptor at Tyr591 in a chemiluminescent ELISA assay. The differences of FLT3-ligand stimulated to untreated results are shown. Values are expressed as mean +/− S.D. of three independent experiments. For further statistical analyses FLT3 mutants were divided into groups of FLT3-WT-like (FLT3-I867S/-V592A), FLT3-TKD (FLT3-D839G/-D835V/-D835Y) and FLT3-ITD (FLT3-ITD1/-ITD2/-ITD3) cell lines. In grouped analysis FLT3-WT expressing cells were significantly different compared to the FLT3-TKD (p = 0.019) but not to FLT3-WT-like cell lines. Groups of FLT3-WT-like, FLT3-TKD and FLT3-ITD cell lines were significant among each other (p≤0.030). (*) indicates significance of grouped analyses and individually compared to FLT3-WT expressing cells. RLU: Relative light units.

Proliferation experiments revealed significant growth advantages for all FLT3 mutants relative to FLT3-WT expressing cells (p≤0.001). FLT3-ITD expressing cells grew completely IL-3 independent, regardless of length and insertion site. Cell lines expressing FLT3-V592A, -I867S, -D839G or -D835V/Y mutations showed a clear gain-of-function phenotype, but differed significantly with respect to their IL-3 independence. Defining the average growth of FLT3-ITD expressing cells as 100%, FLT3-WT, -V592A and -I867S transfected cells displayed growth rates of 2.8%, 7.8% and 8.2%, respectively. FLT3-TKD mutants grew on a range between 14.5% and 42.5% (Figure 2A).

All FLT3 mutants conveyed protection from apoptosis to different degrees (apoptosis ratio of FLT3-WT = 100%). FLT3-V592A and -I867S achieved 59.7% (p = 0.057) and 58.9% (p = 0.059), respectively. FLT3-TKD apoptosis rates ranged between 26.9–35.6% (p≤0.039) and FLT3-ITD cell lines showed apoptosis rates similar to IL-3 controls (13.3–13.7%; p = 0.002) (Figure 2B).

We evaluated the effects of FLT3 ligand (FL) stimulation using Tyr591 phosphorylation as readout. In FLT3-WT, -I867S and -V592A expressing cells a distinctive effect of FL on receptor phosphorylation was seen. FLT3-TKD mutants showed intermediate response , whereas no effect was seen in FLT3-ITD compared to FLT3-WT expressing cells (p≤0.046) (Figure 2C).

Taken together, all FLT3 mutations analyzed in this cell line model expressed a distinct gain-of-function phenotype.

### FLT3 Receptor Mutants Show Distinct Glycosylation and Cell Surface Expression Patterns

**Figure 3:**
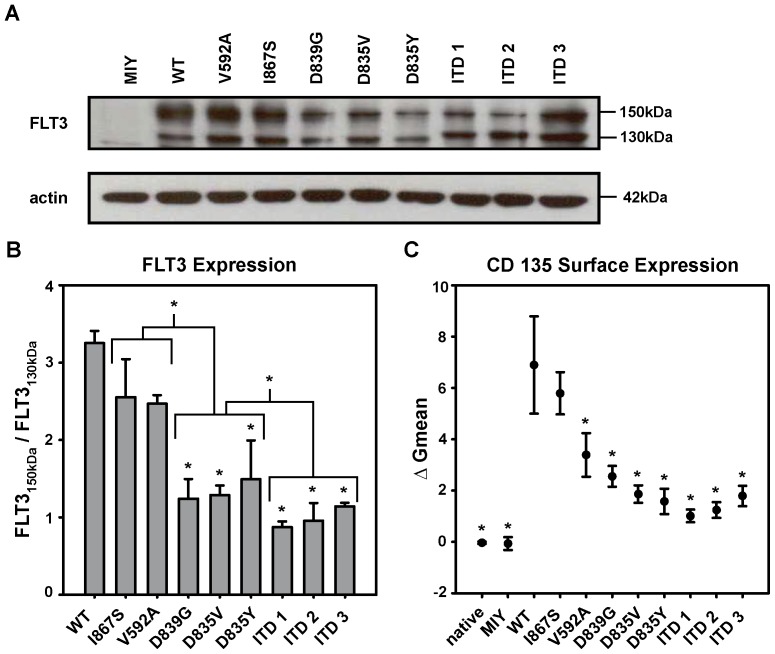
Glycosylation pattern and cell surface expression of FLT3 receptor differ in FLT3 mutants. (A) FLT3 mutants were cultured without IL-3 for 24 hours prior to lysis. One out of three independently representative experiments is shown. (B) To illustrate the expression of the 150/130 kDa forms of FLT3 semi-quantitive analyses of western blot band intensity were performed. Depicted is the ratio of the 150 kDa to the 130 kDa FLT3 form. Values are expressed as mean +/− S.D. of three independent experiments. Ratio of 150 kDa to 130 kDa form of FLT3 was significantly different for each FLT3-TKD (p≤0.009) and each FLT3-ITD (p≤0.002) cell line, but not for FLT3-V592A and -I867S expressing cells compared to FLT3-WT expressing cells. FLT3-WT expressing cells showed a significant differnence to the groups of FLT3-WT-like mutants (p = 0.039), FLT3-TKD mutants (≤0.001) and FLT3-ITD mutants (p≤0.001). Further FLT3-WT like mutants were significantly different compared to the FLT3-TKD group (p≤0.001) and FLT3-TKD mutants were significantly different compared to FLT3-ITD cell lines (p = 0.004). (*) indicates significance of grouped analyses and individually compared to FLT3-WT expressing cells. (C) Ba/F3 cell lines stably expressing the indicated FLT3 constructs were stained with CD-135 antibody and analyzed by flow cytometry. Expression is depicted as difference of geometric mean to isotype control (ΔGmean). Values are expressed as means +/− S.D. of four independent experiments. (*) indicates significance compared to FLT3-WT expressing cells.

To further analyze the differences in cell growth we performed a Western blot assay for the FLT3 protein. As reported before, FLT3 is detectable as two bands, representing the strong glycosylated 150 kDa and weak glycosylated 130 kDa FLT3 species [31]. The FLT3 protein was present in all cell lines, with equal actin controls (Figure 3A). We noted that a strong signal for the 150 kDa-sized FLT3 protein could be detected in FLT3-WT, FLT3-V592A and FLT3-I867S expressing cells in comparison to FLT3-ITD mutants which show a higher proportion of the 130 kDa-sized form. The remaining FLT3-TKD mutation expressing cells fall in between those two extremes (Figure 3B).

In order to evaluate the distribution of the FLT3 receptor on the cell surface, the Ba/F3 cell lines were stained with a CD135 antibody and evaluated using flow cytometry. Difference of geometric mean fluorescence to isotype control antibody showed no significant difference between FLT3-WT and -I867S expressing cells. Interestingly, the cell surface expression of the FLT3 receptor was significantly reduced by a factor of approximately two between FLT3-WT and FLT3-V592A expressing cells (p = 0.029). The cell surface expression of the FLT3 receptor decreased further for FLT3-TKD and FLT3-ITD cell lines (p≤0.029) (Figure 3C). Reduced cell surface expression of FLT3 was directly correlated with the 150/130 kDa ratio.

### FLT3-ITD Mutants Induce STAT5 and FLT3 Tyr591 Phosphorylation

**Figure 4:**
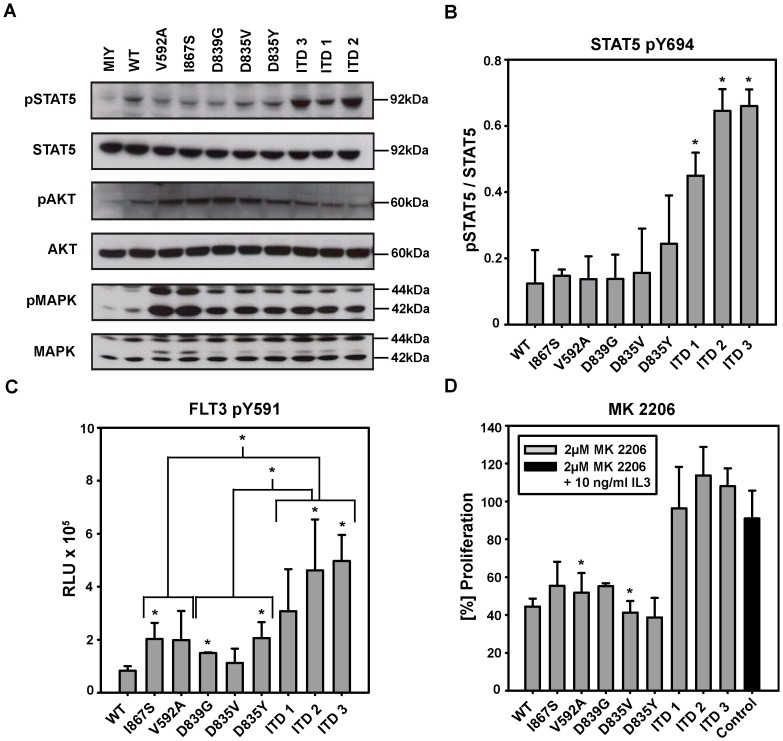
FLT3-ITD mutants induce STAT5 and Tyr 591 phosphorylation whereas FLT3 point mutations rely on AKT and MAPK activation. (A) Ba/F3 cells expressing FLT3 mutations were cultured without IL-3 for 24 hours prior to lysis. Blots were probed against STAT5 pTyr694, AKT pSer473 and MAPK pThr202/pTyr204 stripped and subsequently reprobed against total STAT5, AKT and MAPK. One out of three independent representative experiments is shown. (B) Semi-quantitive analysis of (p)STAT5 band intensity was performed to calculate the ratio between pSTAT5 and total STAT5. Values are expressed as mean +/− S.D. of three independent experiments. (*) indicates significance to FLT3-WT expressing cells. The signal intensity of Ba/F3 MIY expressing cells has been subtracted from FLT3-WT and FLT3 mutant values. (C) Ba/F3 cells expressing FLT3 mutations were cultured without IL-3 for 24 hours prior to lysis. Equal amount of whole cell lysates were used in a chemiluminescent ELISA assay detecting phosphorylation of FLT3 receptor at Tyr591. Values are expressed as mean +/− S.D. of three independent experiments. Grouped analysis of FLT3 mutants revealed significant differences between FLT3-WT expressing cells and FLT3-WT-like cell lines (p = 0.045) as well as FLT3-WT expressing cells and FLT3-ITD cell lines (p = 0.005). Further the group of FLT3-ITD cell lines showed significant differences compared to FLT3-WT-like mutants (p = 0.008) and FLT3-TKD cell lines (p≤0.001). (*) indicates significance of grouped analyses and individually compared to FLT3-WT expressing cells. The signal intensity of Ba/F3 MIY expressing cells has been subtracted from FLT3-WT and FLT3 mutant values. RLU: Relative light units. (D) 4×10^4^ cells/ml stably transduced with indicated constructs, were cultured in presence or absence of 2 µM MK 2206 and/or 10 ng/ml IL-3. Cells were counted after 72 hours by trypan blue exclusion. (*) indicates significant growth reduction by MK 2206 compared to untreated cells. Proliferation with MK 2206 is shown in relation to untreated cells. Control indicates for Ba/F3 MIY expressing cells treated with 2 µM MK 2206 in the presence of IL-3. Values are expressed as mean +/− S.D. of three independent experiments.

To gain insight into the signaling properties of the various FLT3 mutants, we analyzed STAT5 phosphorylation by Western blot (Figure 4A).

STAT5 was only activated in FLT3-ITD expressing cells (p≤0.010), but not in the cell lines expressing FLT3 point mutations. Ba/F3 cells expressing FLT3-ITD1 showed the weakest STAT5 activation in the group of ITD mutations (Figure 4B).

To further confirm the role of STAT5 activation in FLT3 mutants, an ELISA detecting phosphorylation of the FLT3 receptor at Tyr591 was performed. Tyr591 has been described to be critical for the regulative activity of STAT5 and becomes accessible through the conformational changes induced by ITD [32]. All FLT3-ITD mutants induced considerable phosphorylation of Tyr591. Probably due to the small number of values, significance compared to FLT3-WT could be verified for FLT3-ITD2 and -ITD3 (p≤0.027), but not for FLT3-ITD1 expressing cells. FLT3-I867S, -D839G and -D835Y expressing cells exceeded phosphorylation levels of Tyr591 of FLT3-WT expressing cells significantly (p≤0.031) (Figure 4C).

### In Contrast to FLT3-ITD Mutants FLT3 Point Mutation Expressing Cells Rely on AKT and MAPK Signaling and are Inhibited by AKT Inhibitor MK 2206

In Western blot analysis, activation level of AKT for FLT3-I867S, D839G and -D835V expressing cell lines were significantly stronger than in FLT3-WT expressing cells (p≤0.047). FLT3-V592A and FLT3-ITD mutants did not show statistically significant differences of AKT activation levels compared to FLT3-WT expressing cells (Figure 4A, Figure S2). FLT3-D839G, -D835V and -D835Y expressing cells displayed enhanced activation levels of MAPK compared to FLT3-WT expressing cells (p≤0.020). The results were not statistically significant for FLT3-ITD mutants (Figure 4A, Figure S2).

In order to detect a correlation between enhanced AKT signaling and proliferation we treated the FLT3 mutants expressing cell lines with MK 2206, a novel highly selective AKT inhibitor [33]. MK 2206 inhibited growth of FLT3-WT, FLT3-I867S, FLT3-D839G and FLT3-D835Y expressing cell lines, but showed a significant effect on cell growth only for FLT3-V592A (p = 0.024) and -D835V (p = 0.048). FLT3-ITD cell lines were not affected in their growth by MK 2206 (Figure 4D). These data show that AKT and MAPK activation plays an essential role in the activating phenotype of FLT3-non-ITD mutants.

**Figure 5:**
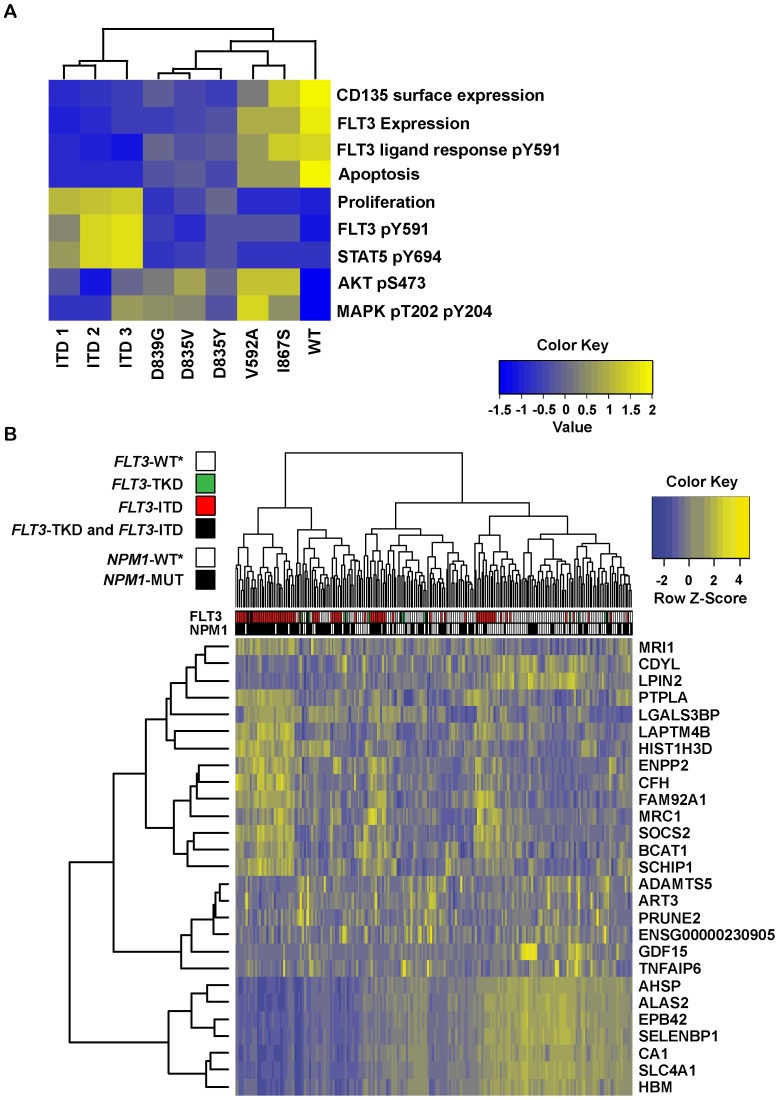
Heatmap of FLT3 signaling and gene expression profiles in AML blast cells. (A) Graphic illustration of FLT3 mutants with respect to biological characteristics according to performed experiments. FLT3 mutants were clustered hierarchically based on similarity and displayed in columns. Experiments were ordered in rows as indicated. (B) Heatmap of probe sets differentially expressed in *FLT3*-ITD, -TKD and -WT CN-AML. The 27 probe sets were selected according to FLT3 and NPM1 mutation status (Table S3) with following characteristics: Log fold change > or <1.5. If no probe set met this criterion, the ten most significant probe sets were selected. Rows: probe sets (n = 27); Columns: patients (n = 213).

The data of the *in vitro* experiments are summarized in a heatmap, visualizing the functional characteristics of all analyzed FLT3 mutants (Figure 5A).

### Gene Expression Profiles Reveal Distinct Differences with Respect to FLT3 Mutation Type

We compared gene expression profiles (GEP) with respect to *FLT3* mutation status (*FLT3*-ITD vs. *FLT3*-TKD). We defined subgroups according to *NPM1* mutation status, due to the correlation with *FLT3*-ITD and influence on gene expression. Out of the GSE37642 data set 76 patients were *FLT3*-ITD positive, 11 patients showed a TKD mutation and six patients had both genetic aberrations. Most patients expressed none of these mutations (*FLT3*-WT*; n = 120).

In differential gene expression analysis, *FLT3*-ITD showed a stronger impact on gene expression than *FLT3*-TKD with respect to *NPM1* mutational status after adjustment for multiple testing (p≤0.05). Compared to *FLT3*-WT* 8573 genes were differentially expressed in FLT3-ITD patients, of those 3048 were up-regulated. Interestingly, when we analyzed the subgroup of patients without an additional *NPM1* mutation only 23 genes were deregulated (19 up-regulated). When *FLT3*-ITD positive patients were compared to the group of *FLT3*-TKD mutations, 709 genes were differentially expressed, of those 143 were up-regulated. In the GEP of patients with *FLT3*-TKD mutations irrespective of *NPM1* status, there were no genes that showed significant differential expression levels compared to *FLT3*-WT* patients (Table S2). However, in both subgroup analysis (*NPM1* mutated and *NPM1*-WT*) *PRUNE2* and *ART3* were significantly higher expressed in patients with *FLT3*-TKD. The genes most prominently differently expressed in *FLT3*-ITD positive patients were *SOCS2*, *ENPP2* and *MRC1*, which were up-regulated. The results for selected genes are presented in a heatmap, showing a heterogeneous profile possibly influenced by additional mutations (Figure 5B). Details of differential gene expression analysis are available upon request.

We used Gene set enrichment analysis (GSEA) to identify differences in signaling pathways related to the *FLT3* status. The comparison of *FLT3*-ITD to *FLT3*-WT* revealed 30 gene sets significant at false discovery rate (FDR) <25% and 24 gene sets significantly enriched at nominal p-value<5%. The comparison of *FLT3*-TKD mutations with *FLT3*-WT* showed 13 gene sets significant at FDR <25% and 15 gene sets significantly enriched at nominal p-value<5%. The gene sets present in both subgroups were apoptosis and glycan structures degradation. The *FLT3*-ITD subgroup showed enrichment of a variety of metabolic pathways, whereas in the group of *FLT3*-TKD cytokine and immunologic pathways including the JAK/STAT signaling pathway were enriched (Table S3).

To verify our *in vitro* data showing differences in STAT5 signaling regarding *FLT3* mutation type we used predefined gene sets implemented in GSEA (STAT5A_01 – 04, STAT5B_01). Since *NPM1* is correlated with *FLT3*-ITD and has significant impact on gene expression, we tried to avoid this confounding variable by analyzing different subgroups regarding *NPM1* mutation status. We were able to demonstrate that predefined STAT5A_02 was significantly enriched in the *NPM1*-WT* subgroup, whereas STAT5A_01 and STAT5B_01 were also significant in the *NPM1*-mutated subgroup comparing *FLT3*-ITD with *FLT3*-WT* [34]. *FLT3*-TKD in comparison to *FLT3*-WT* showed significant enrichment of STAT5A_01 – 04 and STAT5B_01 in the *NPM1*-WT* subgroup (Table 2). This data has to be interpreted with caution but suggests that depending on the type of *FLT3* mutations different transcription factors are activated.

**Table 2:** FLT3-ITD and FLT3-TKD show distinct associations with predefined STAT5 target gene subsets.

(A) Enrichment of predefined STAT5 target genes in FLT3-ITD					
	STAT5A_01	STAT5A_02	STAT5A_03	STAT5A_04	STAT5B_01
No selection regarding *NPM1* mutation *FLT3*-ITD+ (n = 76) vs. *FLT3*-WT* (n = 120)	FDR 29%, p = 0.07	FDR 9%, p = 0.02	n.s.	n.s.	FDR 9%, p = 0.007
**Subgroup A** *NPM1* mutation + *FLT3*-ITD+ (n = 50) vs. *FLT3*-WT* (n = 48)	FDR 27%, p = 0.03	FDR 26%, p = 0.05	n.s.	n.s.	FDR 23%, p = 0.008
**Subgroup B** *NPM1* mutation − *FLT3*-ITD+ (n = 25) vs. *FLT3*-WT* (n = 64)	n.s.	FDR 10%, p = 0.04	n.s.	n.s.	n.s.

(B) Enrichment of predefined STAT5 target genes in FLT3-TKD					
	STAT5A_01	STAT5A_02	STAT5A_03	STAT5A_04	STAT5B_01
No selection regarding *NPM1* mutation *FLT3-*TKD+ (n = 11) vs. *FLT3-*WT* (n = 120)	FDR 32%, p = 0.07	n.s.	FDR 17%, p = 0.01	FDR 30%, p = 0.08	FDR15%, p = 0.007
**Subgroup A** *NPM1* mutation + *FLT3*-TKD+ (n = 6) vs. *FLT3*-WT* (n = 48)	n.s.	n.s.	n.s.	n.s.	n.s.
**Subgroup B** *NPM1* mutation − *FLT3*-TKD− (n = 5) vs. *FLT3*-WT* (n = 64)	FDR 5%, p<0.001	FDR 11%, p = 0.04	FDR 11%, p = 0.04	FDR 3%, p = 0.001	FDR 8%, p<0.001

Analysis of STAT5 target genes in context of FLT3-ITD (A) and FLT3-TKD (B) mutation status in primary AML bone marrow samples. To avoid the interference of NPM1 mutations subgroups were composed, according to NPM1 mutation status. The differences in transcription factor target gene sets (STAT5A_01-04) result from different targeted motifs representing the broad spectrum of potential binding sites. FLT3-ITD was associated in all subgroups with significant enrichment of STAT5A_02 target genes and in no subgroup with enrichment of STAT5A_03 and STAT5A_04 target genes. FLT3-TKD showed a more diverse picture with enrichment of STAT5A_03 and STAT5A_04 target genes in some groups. In FLT3-TKD and NPM1-mutated patients no enrichment of STAT5 target genes could be detected. (+) indicates the presence of a mutation; (−) indicated the absence of a mutation; FDR: false discovery rate; p: nominal p-value (estimates the statistical significance of the enrichment score of the gene set); n.s.: not significant.

## Discussion

The most common *FLT3* mutations are ITD and mutations in the second TKD, which are frequently found in AML but also to a lower frequency in ALL [2], [3]. Other groups have shown the adverse impact of *FLT3*-ITD mutations on OS and RFS in the group of CN-AML which could be confirmed in our study [5], [20]. However, the results for *FLT3*-TKD mutations are inconclusive [4], [5], [7], [19]. Our group found a trend towards a better RFS in *FLT3*-TKD positive patients compared to *FLT3*-WT* cases. However, this was not statistically significant. The *FLT3*-TKD mutations did not have a significant impact on OS or RFS in multivariable Cox regression analysis. The majority of *FLT3*-TKD mutations were lost at relapse, whereas no additional TKD mutations were detectable at relapse. Evaluation of the stability of *FLT3*-TKD mutations in AML patients has so far only been described in very small patient cohorts and represents a superior response to treatment [19], [35].

**Figure 6:**
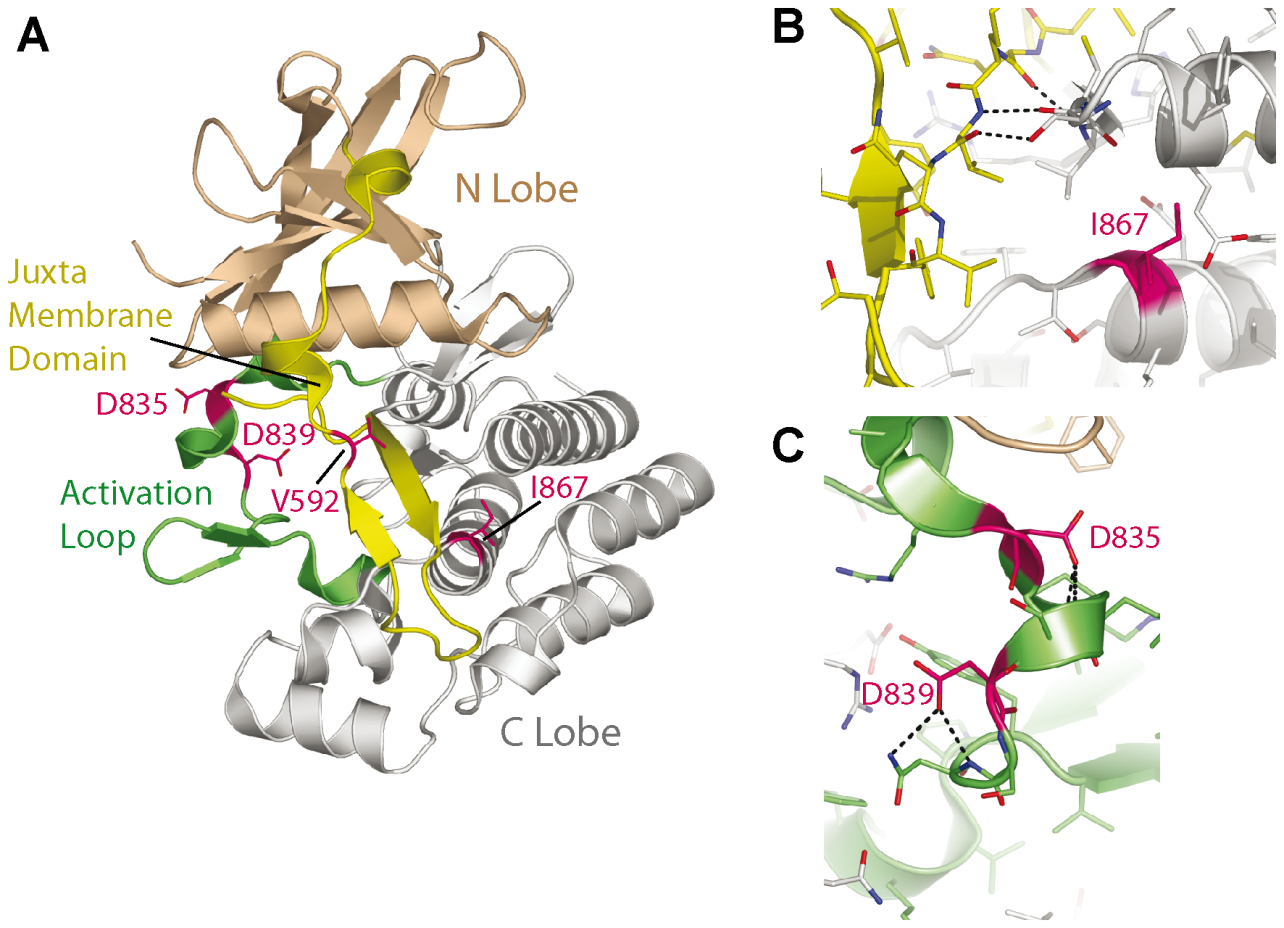
Structural mapping of FLT3-I867 and FLT3-D839. (A) Ribbon representation of the human FLT3 crystal structure (PDB code: 1RJB), with highlighted secondary structure. Mapped mutations are shown as stick models (magenta). (B) Close up view showing the position of I867 (magenta) at the contact face between the C-lobe of the kinase (grey) and juxtamembrane domain (yellow). Notable hydrogen bonds between the juxtamembrane domain and the C-lobe are shown as dashed lines. (C) Close up view showing that D835 and D839 stabilize the fold of the activation loop (green) via hydrogen bonds (dashed lines).

In order to compare the prognostic data with functional aspects, we investigated multiple *FLT3* mutations *in vitro*, including two novel mutations that had not been described before. The point mutation at AA 839 has been found in AML patients before and is similar to known *FLT3* mutations of the second TKD [36], [37]. Not only do *FLT3* point mutations cluster at AA 835, but alterations of AA 834, 836, 840, 841 and 842 have been described before [4], [9]–[13], [17], [37]–[39]. The corresponding positions were analyzed on the crystal structure of human FLT3 (Figure 6; PDB code: 1RJB; [6]). D839 like D835 maps to the activation loop of FLT3, stabilizing its conformation in the autoinhibited state. FLT3-D839G and -D835Y presumably lead to a more flexible activation loop and consequently higher propensity for tyrosine phosphorylation (Figure 6). The corresponding amino acid (D820) of the homologous c-KIT receptor has been described to be mutated in mast cell related diseases, gastrointestinal stromal tumors, haematologic and lymphoid malignancies as well as seminomas [40]. This suggests an aberrant activity of this FLT3 mutant. Furthermore, there are reports of point mutations in other regions of the receptor [9], [10], [37], [39]. The newly identified FLT3-I867S mutation is located directly at the beginning of an alpha helix and is part of a hydrophobic interaction site that stabilizes the helical bundle that forms the C-lobe of the FLT3 kinase domain. A serine at this position likely destabilizes the surface region of the helical bundle. This surface region is an important binding site for the JM domain, which forms several hydrogen bonds as well as hydrophobic interactions with this region (Figure 6). I867S presumably alters the fold or stability around the interaction site to the JM domain and leads to a reduced affinity between JM domain and C-lobe. As a consequence, I867S could reduce the autoinhibition of FLT3 and lead to an increased activation.

For functional evaluation we analyzed proliferation, apoptosis, receptor alteration and signaling *in vitro*. In contrast to prior findings, the various mutations did not present themselves functionally as two distinct groups of mutations, one as drivers – resulting in a completely transformed phenotype – and the second as silent passengers – without influence on cell growth and signaling [17]. The investigated *FLT3* mutations were all gain of function mutations that presented a continuous spectrum of receptor activation *in vitro*. Our results showed gradual growth advantages for FLT3 point mutations in proliferation and apoptosis experiments, rather than an on/off behavior. FLT3-ITD mutants presented maximal aberrant activity as reported before, in contrast FLT3 point mutation expressing cells differed clearly from FLT3-WT but also from FLT3-ITD expressing cells [41].

Interestingly, the capacity for additional stimulation by FLT3 ligand (FL) was inversely proportional to receptor phosphorylation levels. This might be due to the fact that FLT3 mutants result in different subcellular localizations of the receptor. This is also represented in the proportional change of expression of the mature 150 kDa isoform expressed on the cell surface to the less glycosilated 130 kDa isoform of FLT3 protein located inside the cell [31], [42]. Stronger cytokine-independent proliferation and weaker FL response was associated with higher amount of intracellular localization of the receptor. In contrast, FLT3-WT receptor was mainly found on the cell surface and showed a clear response to FL. Again those effects were on a continuum within the group of FLT3 mutants representing the diverse activating potential of FLT3 mutations. FLT3 receptors in ITD expressing cells were mainly located intracellular and non-responsive to FL treatment. The localization of the FLT3-ITD in the endoplasmic reticulum seems to be a major factor in compartment-specific activation of STAT5 [31], [42]. The analysis of signaling revealed a distinct activation of STAT5 by FLT3-ITD mutants, as reported before [41], [43]–[46]. STAT5 is directly or SRC-dependently activated in FLT3-ITD expressing cells *in vitro* via tyrosine residues 589 and 591 [47]–[49]. Accordingly, enhanced phosphorylation of Y591 was found in FLT3-ITD expressing cells in contrast to FLT3-WT expressing cells. Interestingly, also FLT3-I867S, -D839G and -D835Y expressing cells showed phosphorylation of Y591, indicating additional functions of this residue or undetected STAT5 activation. Y591 was identified as a docking site for suppressor of cytokine signaling 6 (*SOCS6*), Lnk, an adaptor protein with negative regulator influence on FLT3 and as interaction site of c-CBL, inducing degradation of the FLT3 receptor [26], [50], [51].

We used predefined STAT5 target gene sets for the evaluation of our GEP analysis to demonstrate a potential influence of *FLT3* mutations on STAT5 activity in primary AML cells. We were able to show differences in STAT5 target gene expression between *FLT3*-ITD and -TKD with distinct characteristics. These results have to be interpreted carefully due to the highly complex role of transcription factors and many unknown variables. To account for this, we tried to adjust our analysis for additional well known mutations in AML by defining subgroups. The reproducibility of these results indicates a significant role but diverging pathways and targets of *FLT3* mutations in STAT5 activity.

AKT and MAPK are signaling pathways of the membrane-bound FLT3-WT receptor [1], [41], [52]. Our Western blot analysis revealed an activation of MAPK and AKT in FLT3 point mutation expressing cells in contrast to FLT3-WT and FLT3-ITD expressing cells. Accordingly, only FLT3 point mutation but not FLT3-ITD expressing cells showed growth inhibition after treatment with the AKT pathway inhibitor MK 2206, indicating aberrantly enhanced activation of this pathway. There are differing reports of AKT and MAPK activation by FLT3-ITD mutants [43]–[46].

Consistent with our *in vitro* analysis we were able to detect differences in the gene expression with respect to *FLT3* mutation status. Our analysis of 213 CN-AML patients was able to validate a distinct gene expression profile especially in case of *FLT3*-ITD, but also *FLT3*-TKD. Genes that were highly significantly differentially expressed in our analysis like *SOCS2* and *ENPP2* in the ITD subgroup have also been described by other groups recently [53], [54]. FLT3-TKD positive patients showed an association with higher *PRUNE2* and *ART3* expression levels which has not been described before. In an earlier study, gene expression levels of patients with newly diagnosed CN-AML showed distinct differences in gene expression profiles with respect to *FLT3*-ITD and *FLT3*-TKD mutation [55]. This analysis did not exclude a potential influence of *NPM1* mutations, which are highly significantly associated with *FLT3* mutations, and was conducted in a relatively small patient cohort. This might account for the very minor overlap with our data.

This study is the first comprehensive work evaluating clinical data with respect to prognosis and gene expression as well as comparative *in vitro* analysis of multiple *FLT3* mutations. Our results strengthen the evidence that *FLT3* mutations have varying activating potential and reject the strict division into driver and passenger mutations [17], [19]. Although not every single mutation might affect the prognosis and outcome of AML, all functionally characterized mutants showed a gain-of-function phenotype *in vitro*. Therefore FLT3 point mutations can contribute to leukemogenesis and are thus potential targets for therapeutic interventions especially with regard to tyrosine kinase inhibitor resistance [14]–[16].

## Supporting Information

Figure S1 Sequence and location of FLT3 mutations analyzed in this study.
(TIF)

Figure S2 FLT3 point mutation expressing cells show AKT and MAPK phosphorylation in contrast to FLT3-ITD cell lines. Ba/F3 cells expressing FLT3 mutations were cultured without IL-3 supplement for 24 hours prior to lysis. Blots were probed against (A) AKT pS473 and (B) MAPK pThr202/Tyr204 stripped and subsequently reprobed against total (A) AKT and (B) MAPK. Semi-quantitive analysis of (p)AKT and (p) MAPK band intensity was performed to calculate the ratio between (A) pAKT and AKT as well as (B) pMAPK and MAPK. Values are expressed as mean +/− S.D. of three independent experiments. (*) indicates significance to FLT3-WT expressing cells. The signal intensity of Ba/F3 MIY expressing cells has been subtracted from FLT3-WT and FLT3 mutant values.
(TIF)

Table S1 Patient characteristics. Patient characteristics of 213 CN-AML patients included in GSE37642. All patients were enrolled in the AMLCG-99 trial and received intensive induction treatment.
(DOCX)

Table S2 Differential gene expression analysis of *FLT3* mutations in AML patients. Differential gene expression analysis of *FLT3*-ITD and -TKD mutations with respect to *NPM1* mutation status. Only genes significant at p≤0.05 after adjustment for multiple testing are displayed.
(DOCX)

Table S3 Comparison of signaling pathways according to *FLT3* mutation status using GSEA. GSEA analysis of *FLT3*-ITD (A) and -TKD (B) with *FLT3*-WT* using the “c2kegg” gene sets. Only gene sets with FDR <25% are displayed. ES: enrichment score; NES: nominal enrichment score; NOM p-val: nominal p-value; FDR q-val: false discovery rate.
(DOCX)
