# Supplementary figures and images for: Activating FLT3 Mutants Show Distinct Gain-of-Function Phenotypes In Vitro and a Characteristic Signaling Pathway Profile Associated with Prognosis in Acute Myeloid Leukemia
Source: PLoS One. 2014 Mar 7;9(3):e89560. doi: 10.1371/journal.pone.0089560 (PMC3946485; doi:10.1371/journal.pone.0089560)

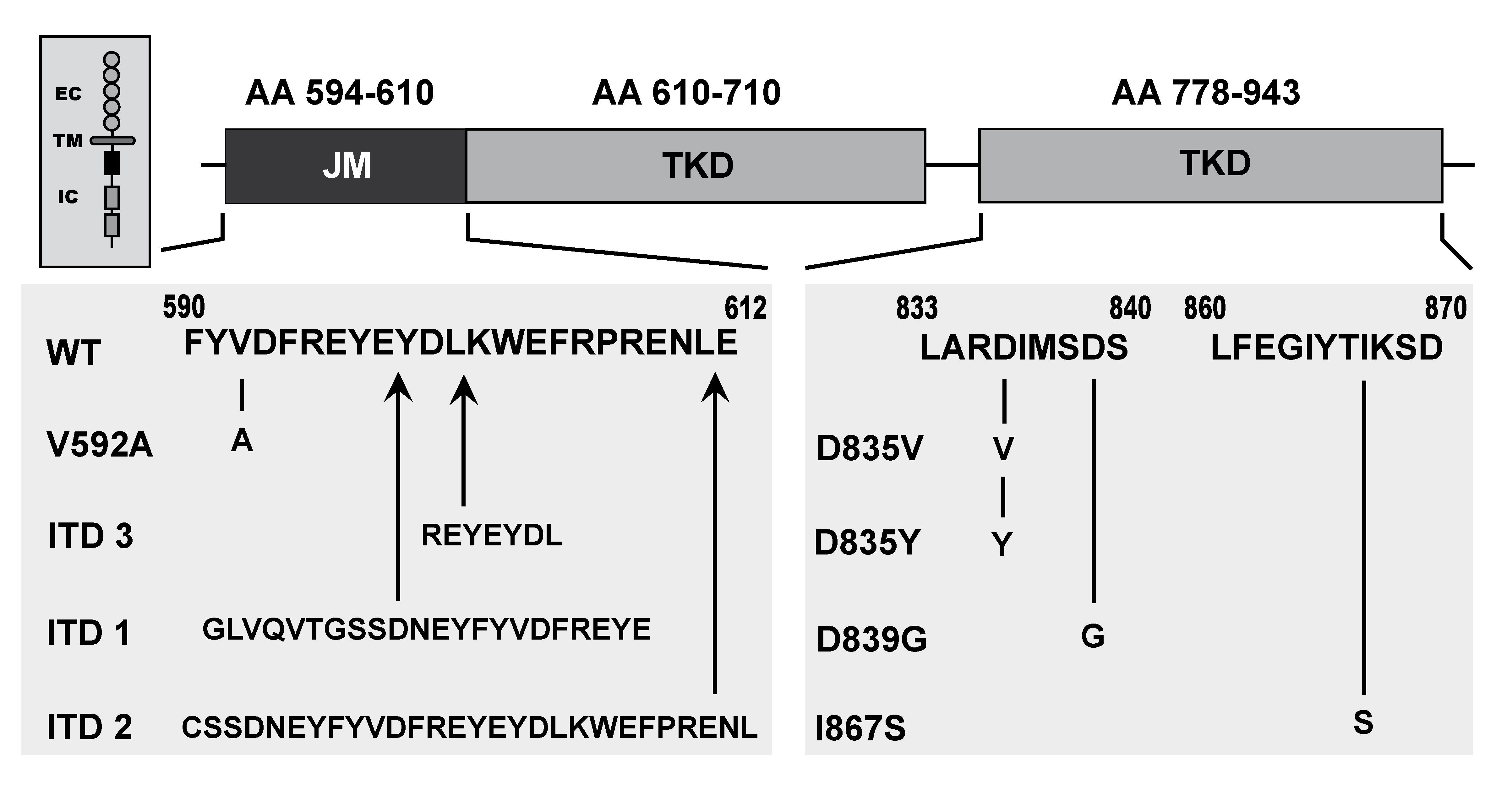

Supplement: Figure S1 — Sequence and location of FLT3 mutations analyzed in this study. (TIF) [file pone.0089560.s001.tif]

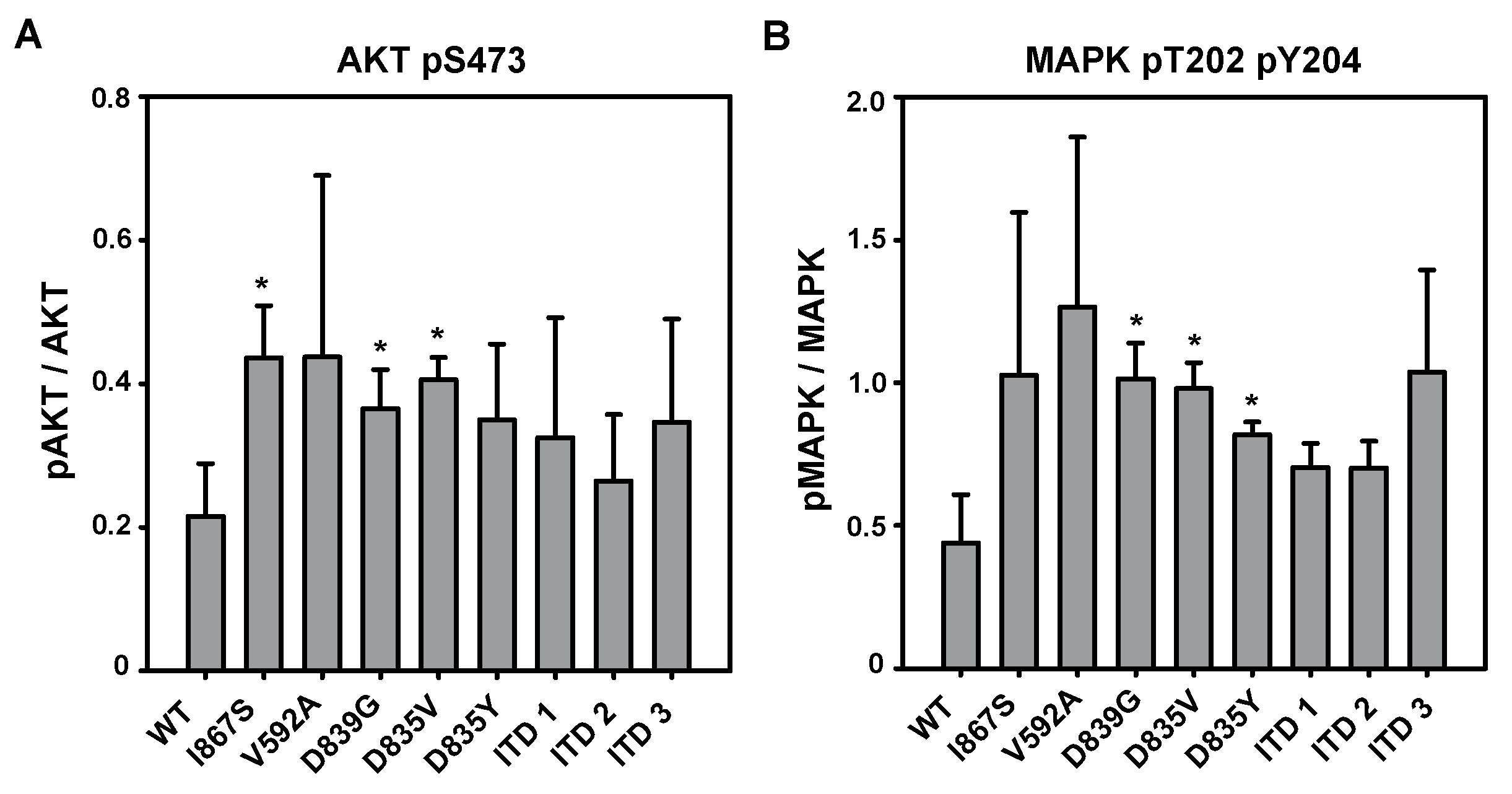

Supplement: Figure S2 — FLT3 point mutation expressing cells show AKT and MAPK phosphorylation in contrast to FLT3-ITD cell lines. Ba/F3 cells expressing FLT3 mutations were cultured without IL-3 supplement for 24 hours prior to lysis. Blots were probed against (A) AKT pS473 and (B) MAPK pThr202/Tyr204 stripped and subsequently reprobed against total (A) AKT and (B) MAPK. Semi-quantitive analysis of (p)AKT and (p) MAPK band intensity was performed to calculate the ratio between (A) pAKT and AKT as well as (B) pMAPK and MAPK. Values are expressed as mean +/− S.D. of three independent experiments. (*) indicates significance to FLT3-WT expressing cells. The signal intensity of Ba/F3 MIY expressing cells has been subtracted from FLT3-WT and FLT3 mutant values. (TIF) [file pone.0089560.s002.tif]
