# Supplementary material for: Activating FLT3 Mutants Show Distinct Gain-of-Function Phenotypes In Vitro and a Characteristic Signaling Pathway Profile Associated with Prognosis in Acute Myeloid Leukemia
Source: PLoS One. 2014 Mar 7;9(3):e89560. doi: 10.1371/journal.pone.0089560 (PMC3946485; doi:10.1371/journal.pone.0089560)
Supplement: Table S1 — Patient characteristics. Patient characteristics of 213 CN-AML patients included in GSE37642. All patients were enrolled in the AMLCG-99 trial and received intensive induction treatment. (DOCX) [file pone.0089560.s003.docx]

| **Variable** | **FLT3-WT*** | **FLT3-TKD** | **FLT3-ITD** | **FLT3- ITD and FLT3-TKD** | **p-value** |
| --- | --- | --- | --- | --- | --- |
| No. of patients | 120 | 11 | 76 | 6 |  |
| Median age, years (range) | 61 (18-83) | 57 (43-75) | 55.5 (20-85) | 55 (47-60) | n.s. |
| Male sex, no. (%) | 53 (44.2) | 8 (72.7) | 29 (38.2) | 1 (16.7) | n.s. |
| White-cell count, G/l, median (range) | 15.3 (0.8-280) | 22.5 (1-100) | 47.7 (0.1-486) | 70 (13.9-123) | <.001 |
| Hemoglobin, g/dl, median (range) | 9.2 (5.3-14.7) | 9 (4.9-11.5) | 9.1 (4.2-14.2) | 10.1 (8.2-12.6) | n.s |
| Platelet count, G/l ,median (range) | 60 (7-301) | 64 (18-160) | 56 (1-471) | 55.5 (20-113) | n.s |
| LDH (U/l), median (range) | 415 (132-4296) | 406 (156-753) | 727 (159-2814) | 535 (331-1182) | <.001 |
| Bone marrow blasts, %, median (range) | 80 (10-100) | 90 (30-100) | 90 (20-100) | 75 (64-97) | n.s |
| Performance Status (ECOG) ≥ 2 (%) | 7 (5.9) | 0 | 8 (11.3) | 0 | n.s. |
| *de novo* AML (%) | 105 (87.5) | 11 (100) | 64 (84.2) | 6 (100) | n.s. |
| *NPM1* mut., no. (%) | 48 (42.9) | 6 (54.5) | 50 (66.7) | 5 (83.3) | .006 |
| *MLL*-PTD, no. (%) | 15 (13.2) | 2 (20) | 5 (6.7) | 0 (0) | n.s. |
| mo*CEBPA*, no. (%) | 6 (5.8) | 0 (0) | 4 (6) | 1 (20) | n.s. |
| bi*CEBPA*, no. (%) | 9 (8.7) | 0 (0) | 1 (1.5) | 0 () | n.s. |
| Complete remission, no. (%) | 76 (63.3) | 8 (72.7) | 47 (61.8) | 3 (50) | n.s. |
| Deceased, no. (%) | 77 (64.2) | 7 (63.6) | 61 (80.3) | 5 (83.3) | .04 |

**Table S1:**
