# Supplementary material for: Activating FLT3 Mutants Show Distinct Gain-of-Function Phenotypes In Vitro and a Characteristic Signaling Pathway Profile Associated with Prognosis in Acute Myeloid Leukemia
Source: PLoS One. 2014 Mar 7;9(3):e89560. doi: 10.1371/journal.pone.0089560 (PMC3946485; doi:10.1371/journal.pone.0089560)
Supplement: Table S2 — Differential gene expression analysis of FLT3 mutations in AML patients. Differential gene expression analysis of FLT3-ITD and -TKD mutations with respect to NPM1 mutation status. Only genes significant at p≤0.05 after adjustment for multiple testing are displayed. (DOCX) [file pone.0089560.s004.docx]

**Table S2:**

|  | ***NPM1*mut.** | ***NPM1*-WT*** | ***NPM1*mut. and -WT*** |
| --- | --- | --- | --- |
| ***FLT3*-ITD vs. WT*** | n=50 vs. n=48 deregulated: 3833 up‑regulated: 1976 down‑regulated: 1857 | n=25 vs. n=64 deregulated: 23 up‑regulated: 19 down‑regulated: 4 | n=76 vs. n=120, deregulated: 8573, up-regulated: 3048, down-regulated: 5525 |
| ***FLT3*-TKD vs. WT*** | n=6 vs. n=48 deregulated: 2 up‑regulated: 2 down‑regulated: 0 | n=5 vs. n=64 deregulated: 2 up‑regulated: 2 down‑regulated: 0 | n=11 vs. n=120  deregulated: 0  up-regulated: 0  down-regulated: 0 |
| ***FLT3*-ITD vs. TKD** | n=50 vs. n=6 deregulated: 10 up‑regulated: 3 down‑regulated: 7 | n=25 vs. n=5 deregulated: 1624 up‑regulated: 141 down‑regulated: 1483 | n=76 vs. n=11 deregulated: 709 up‑regulated: 143 down‑regulated: 566 |
