# Supplementary material for: Activating FLT3 Mutants Show Distinct Gain-of-Function Phenotypes In Vitro and a Characteristic Signaling Pathway Profile Associated with Prognosis in Acute Myeloid Leukemia
Source: PLoS One. 2014 Mar 7;9(3):e89560. doi: 10.1371/journal.pone.0089560 (PMC3946485; doi:10.1371/journal.pone.0089560)
Supplement: Table S3 — Comparison of signaling pathways according to FLT3 mutation status using GSEA. GSEA analysis of FLT3-ITD (A) and -TKD (B) with FLT3-WT* using the “c2kegg” gene sets. Only gene sets with FDR <25% are displayed. ES: enrichment score; NES: nominal enrichment score; NOM p-val: nominal p-value; FDR q-val: false discovery rate. (DOCX) [file pone.0089560.s005.docx]

| **A** | | | | |
| --- | --- | --- | --- | --- |
| **NAME** | **ES** | **NES** | **NOM p-val** | **FDR q-val** |
| HSA00190_OXIDATIVE_PHOSPHORYLATION | 0.574 | 2.558 | <0.001 | <0.001 |
| HSA00280_VALINE_LEUCINE_AND_ISOLEUCINE_DEGRADATION | 0.660 | 2.512 | <0.001 | <0.001 |
| HSA00970_AMINOACYL_TRNA_BIOSYNTHESIS | 0.678 | 2.429 | <0.001 | <0.001 |
| HSA00020_CITRATE_CYCLE | 0.665 | 2.278 | <0.001 | <0.001 |
| HSA00640_PROPANOATE_METABOLISM | 0.664 | 2.277 | <0.001 | <0.001 |
| HSA03050_PROTEASOME | 0.686 | 2.198 | <0.001 | <0.001 |
| HSA03010_RIBOSOME | 0.562 | 2.185 | <0.001 | 0.001 |
| HSA03020_RNA_POLYMERASE | 0.608 | 1.938 | <0.001 | 0.010 |
| HSA00100_BIOSYNTHESIS_OF_STEROIDS | 0.601 | 1.937 | <0.001 | 0.009 |
| HSA00790_FOLATE_BIOSYNTHESIS | 0.487 | 1.797 | 0.008 | 0.028 |
| HSA00071_FATTY_ACID_METABOLISM | 0.470 | 1.783 | 0.004 | 0.030 |
| HSA00410_BETA_ALANINE_METABOLISM | 0.533 | 1.747 | 0.017 | 0.038 |
| HSA00770_PANTOTHENATE_AND_COA_BIOSYNTHESIS | 0.600 | 1.729 | 0.012 | 0.041 |
| HSA00620_PYRUVATE_METABOLISM | 0.459 | 1.701 | 0.012 | 0.048 |
| HSA01032_GLYCAN_STRUCTURES_DEGRADATION | 0.505 | 1.673 | 0.017 | 0.054 |
| HSA00565_ETHER_LIPID_METABOLISM | 0.487 | 1.650 | 0.016 | 0.060 |
| HSA04662_B_CELL_RECEPTOR_SIGNALING_PATHWAY | 0.397 | 1.643 | 0.002 | 0.060 |
| HSA05040_HUNTINGTONS_DISEASE | 0.467 | 1.583 | 0.023 | 0.089 |
| HSA00650_BUTANOATE_METABOLISM | 0.411 | 1.544 | 0.039 | 0.110 |
| HSA04210_APOPTOSIS | 0.363 | 1.528 | 0.012 | 0.117 |
| HSA00240_PYRIMIDINE_METABOLISM | 0.359 | 1.513 | 0.016 | 0.124 |
| HSA00310_LYSINE_DEGRADATION | 0.403 | 1.511 | 0.038 | 0.119 |
| HSA00670_ONE_CARBON_POOL_BY_FOLATE | 0.511 | 1.507 | 0.062 | 0.118 |
| HSA00510_N_GLYCAN_BIOSYNTHESIS | 0.415 | 1.488 | 0.037 | 0.127 |
| HSA00230_PURINE_METABOLISM | 0.315 | 1.446 | 0.016 | 0.159 |
| HSA00563_GLYCOSYLPHOSPHATIDYLINOSITOL_ANCHOR_BIOSYNTHESIS | 0.452 | 1.422 | 0.072 | 0.177 |
| HSA00051_FRUCTOSE_AND_MANNOSE_METABOLISM | 0.390 | 1.422 | 0.082 | 0.171 |
| HSA00252_ALANINE_AND_ASPARTATE_METABOLISM | 0.390 | 1.414 | 0.071 | 0.173 |
| HSA00380_TRYPTOPHAN_METABOLISM | 0.356 | 1.376 | 0.061 | 0.211 |
| HSA00500_STARCH_AND_SUCROSE_METABOLISM | 0.333 | 1.352 | 0.067 | 0.234 |
| **B** | | | | |
| HSA04060_CYTOKINE_CYTOKINE_RECEPTOR_INTERACTION | 0.556 | 2.332 | <0.001 | <0.001 |
| HSA04620_TOLL_LIKE_RECEPTOR_SIGNALING_PATHWAY | 0.617 | 2.320 | <0.001 | <0.001 |
| HSA04140_REGULATION_OF_AUTOPHAGY | 0.709 | 2.046 | <0.001 | <0.001 |
| HSA04630_JAK_STAT_SIGNALING_PATHWAY | 0.473 | 1.865 | <0.001 | 0.013 |
| HSA04650_NATURAL_KILLER_CELL_MEDIATED_CYTOTOXICITY | 0.492 | 1.863 | <0.001 | 0.011 |
| HSA04940_TYPE_I_DIABETES_MELLITUS | 0.632 | 1.791 | <0.001 | 0.022 |
| HSA01032_GLYCAN_STRUCTURES_DEGRADATION | 0.572 | 1.698 | 0.008 | 0.052 |
| HSA04612_ANTIGEN_PROCESSING_AND_PRESENTATION | 0.515 | 1.642 | 0.008 | 0.081 |
| HSA04640_HEMATOPOIETIC_CELL_LINEAGE | 0.434 | 1.588 | 0.006 | 0.125 |
| HSA00020_CITRATE_CYCLE | 0.534 | 1.583 | 0.028 | 0.118 |
| HSA00531_GLYCOSAMINOGLYCAN_DEGRADATION | 0.574 | 1.531 | 0.046 | 0.169 |
| HSA00340_HISTIDINE_METABOLISM | 0.469 | 1.476 | 0.031 | 0.236 |
| HSA04210_APOPTOSIS | 0.403 | 1.475 | 0.021 | 0.220 |
| HSA00534_HEPARAN_SULFATE_BIOSYNTHESIS | 0.538 | 1.449 | 0.060 | 0.251 |

**Table S3:**
